# Clinical Characteristics and Outcomes of Cytomegalovirus DNAemia in Non-HIV-Infected and Non-Transplant Patients: A Propensity Score-Matched Analysis

**DOI:** 10.3390/pathogens15050492

**Published:** 2026-05-01

**Authors:** Ixchel Salter, Michaele-Francesco Corbisiero, Daniel B. Chastain, Chia-Yu Chiu, Leland Shapiro, Jose G. Montoya, Raymund R. Razonable, Andrés F. Henao-Martínez

**Affiliations:** 1 Department of Medicine, Division of Infectious Diseases, University of Colorado Denver, Aurora, CO 80045, USA; michaelefrancesco.corbisiero@cuanschutz.edu (M.-F.C.); chia-yu.chiu@cuanschutz.edu (C.-Y.C.); leland.shapiro@cuanschutz.edu (L.S.); 2 Department of Clinical and Administrative Pharmacy, University of Georgia College of Pharmacy, Albany, GA 31701, USA; daniel.chastain@uga.edu; 3 Dr. Jack S. Remington Laboratory for Specialty Diagnostics, Palo Alto, CA 94301, USA; jose.montoya2@sutterhealth.org; 4 Division of Public Health, Infectious Diseases and Occupational Medicine, Mayo Clinic, Rochester, MN 55905, USA; razonable.raymund@mayo.edu

**Keywords:** cytomegalovirus, CMV DNAemia, immunocompetent, HIV-negative, non-transplant, all-cause mortality, propensity score matching

## Abstract

Cytomegalovirus (CMV) establishes lifelong latency following primary infection and can reactivate to cause severe illness in immunocompromised hosts. CMV DNAemia in non-HIV-infected, non-solid organ/bone marrow transplant (NHNT) hosts is poorly characterized, with limited clinical insights. We aim to describe the clinical presentation, prognostic indicators, and outcomes of CMV DNAemia among NHNT patients. We used the TriNetX international patient database to identify adult patients diagnosed with CMV DNAemia from 2016 until March 2023. We evaluated hospitalization, intensive care unit (ICU) level care, and all-cause mortality at 30 days and 1 year. We also completed a post-propensity score analysis comparing clinical characteristics of survivors versus non-survivors at 90 days. We identified 1123 NHNT patients with CMV DNAemia, most of whom had neoplasms (63%). Venous thromboembolism occurred in 31% of the population. The 30-day hospitalization and all-cause mortality rates were 35% and 14%, respectively. After propensity score matching and Bonferroni correction, weakness, purpura, acute respiratory failure, malnutrition, encephalopathy, and hypotension were associated with increased 90-day all-cause mortality. NHNT patients with CMV DNAemia carry a substantial morbidity and all-cause mortality. Further studies are warranted to clarify whether CMV DNAemia is a causative factor or an incidental finding in this population.

## 1. Introduction

Cytomegalovirus (CMV) is a ubiquitous betaherpesvirus that establishes lifelong latency following primary infection. Respiratory droplets frequently transmit it, direct contact with bodily fluids, or maternal–fetal transmission, with worldwide seroprevalence (indicative of prior infection) of 30–70%, varying by geographic region and socioeconomic conditions [1]. Immunocompetent hosts with initial CMV infection generally present with nonspecific mononucleosis-like syndromes characterized by fever, malaise, lymphocytosis, and mild hepatitis, which are typically self-limited [1,2]. Following primary infection, CMV persists in a latent state within myeloid progenitor cells and can reactivate when host immune surveillance is impaired [1,2]. CMV DNA indicates subsequent CMV reactivation in blood (DNAemia), reflecting active viral replication.

CMV reactivation can cause clinically significant end-organ disease—including pneumonia, colitis, encephalitis, retinitis, and, less commonly, hepatitis—in immunocompromised hosts [3,4]. CMV pneumonia, in particular, is among the most severe manifestations and carries a high mortality rate, especially in hematopoietic cell transplant (HCT) recipients, where historical mortality rates approached 70% before the advent of modern antiviral strategies [5]. Populations at risk for clinically significant CMV reactivation extend well beyond those with advanced HIV infection or solid organ transplant recipients. These include HCT recipients, patients with primary immunodeficiency disorders, those with hematologic malignancies receiving intensive chemotherapy or novel immunotherapies (such as chimeric antigen receptor [CAR] T-cell therapy, bispecific T-cell-engaging antibodies, or phosphoinositide 3-kinase [PI3K] inhibitors), and patients with autoimmune or inflammatory conditions receiving immunosuppressive therapy (e.g., high-dose glucocorticoids, calcineurin inhibitors, or biologic agents) [6,7]. The ECIL 7 guidelines specifically identify patients with lymphoid malignancies, those receiving T-cell-suppressive therapy with purine analogs, and patients receiving alemtuzumab as non-HSCT patient groups at the highest risk for CMV-associated complications [8]. More recently, the ECIL 10 recommendations have expanded the scope of at-risk populations to include patients treated with CAR T cells and T-cell-engaging antibodies, in whom CMV reactivation rates of 17–56% have been reported [9]. Additionally, critical illness itself can impair host defense mechanisms sufficiently to permit CMV reactivation, even in patients without a traditional immunocompromising condition [4,10,11,12,13]. Prospective studies in ICU populations have demonstrated CMV reactivation rates of 13–35% among seropositive, immunocompetent critically ill patients, with reactivation independently associated with prolonged hospitalization and increased mortality [10,11,12].

Despite this expanding understanding of CMV reactivation across diverse immunocompromised populations, the diagnosis and clinical significance of CMV DNAemia in non-HIV-infected, non-solid organ/bone marrow transplant (NHNT) populations remains poorly understood, given its relative rarity and nonspecific or subclinical presentation. CMV seropositivity has been associated with increased mortality in geriatric populations, but it remains unclear whether this is a causative or incidental finding [14]. There is limited insight regarding comorbidities, symptoms, laboratory findings, and medications associated with poorer prognosis in NHNT populations. The fundamental question of whether CMV DNAemia in these patients represents a pathogenic driver of adverse outcomes or merely a bystander marker of severe immunosuppression and critical illness remains unresolved [13]. This study aims to identify critical clinical features associated with 90-day all-cause mortality in NHNT patients diagnosed with active CMV DNAemia, thereby contributing to the growing body of evidence characterizing CMV reactivation outside of traditional transplant and HIV settings.

## 2. Materials and Methods

### 2.1. Global Federated Research Network

Data for this study were gathered from the TriNetX global research database from January 2016 until March 2023. We defined CMV DNAemia as the presence of ICD-10 code B25 with a CMV viral load ≥5000 IU/mL based on logical observation identifiers, names, and codes (LOINCs) (Supplementary Materials S1 and S2). The level of CMV DNAemia is available in TriNetX. However, it is not specific to CMV disease. Importantly, CMV DNAemia does not equate to CMV end-organ disease, which requires histopathological confirmation or compatible clinical and virologic criteria for definitive diagnosis. Current consensus definitions, including those from the ECIL 7 and the Cytomegalovirus Drug Development Forum, distinguish between CMV infection (detection of CMV replication markers), clinically significant CMV infection (CMV replication warranting preemptive therapy), and CMV disease (end-organ involvement with histopathologic or clinical confirmation) [9]. Our study captures CMV infection as defined by DNAemia, not CMV disease.

Preemptive treatment of patients with asymptomatic CMV reactivation outside the transplant setting is not a universal standard of care, and treatment initiation is generally guided by a combination of patient presentation, quantitative nucleic acid testing (NAT)-based assessment, and histopathological confirmation. Significant variation exists in the correlation between viral load and disease presentation; however, elevated viral loads have been associated with increased disease burden and mortality in patients with confirmed CMV disease. Therefore, without specific data linking patient presentation to viral load trends, we selected 5000 IU/mL as the threshold for serum or plasma to reduce the risk of capturing asymptomatic low-level CMV reactivation. This threshold was chosen pragmatically, as no universally accepted viral load cutoff directly correlates with CMV disease across all patient populations [5]. TriNetX normalizes different reported CMV viremia values. Patients living with HIV or solid organ/bone marrow transplant recipients were excluded based on ICD-10 codes.

The TriNetX platform provides continuously updated, de-identified patient data from the electronic medical records of over 100 million patients across more than 80 medical centers. Our group has published several reports using the same database and methodology [15,16]. Extracted data are structured or unstructured by Natural Language Processing Technology. Most participating healthcare organizations (HCOs) are large academic medical institutions with inpatient and outpatient facilities spanning multiple countries. The data they provide represent the entire patient population at the HCO. Most give an average of seven years of historical data. TriNetX receives data directly from an HCO research repository into the TriNetX environment, or the HCO sends TriNetX data extracts in the form of CSV files coded in the TriNetX Data Dictionary. HCO and other data providers update information at various times, with over 80% refreshing in one-, two-, or four-week intervals. The average lag time for an HCO’s source data to update information is one month. TriNetX maps the data to a standard, controlled set of clinical terminologies and transforms it into a proprietary data model. This transformation process includes extensive data quality assessment and cleaning, rejecting records that do not meet TriNetX quality standards.

### 2.2. Study Design and Population

The NHNT CMV DNAemia cohort (n = 1123) was established using ICD-10 codes and laboratory values (Supplementary Tables S1–S4). The index event was identified as a CMV DNAemia diagnosis coinciding with an elevated viral load (Supplementary Materials S1). We captured demographics, comorbidities, medications, laboratory values recorded within 90 days of CMV DNAemia documentation, and symptoms noted within 30 days of the index event. In patients with multiple episodes of CMV DNAemia, only the initial laboratory values were recorded. Laboratory results were not trended over time; CMV serostatus and histopathology reports were not available. Clinical characteristics were compared between survivors (n = 889) and non-survivors (n = 234) (Supplementary Table S5). Importantly, the temporal relationship between the index CMV DNAemia event and the captured symptoms, laboratory values, and medication exposures cannot be reliably determined within the TriNetX platform. Symptoms were captured within 30 days and laboratory values within 90 days of CMV DNAemia documentation, but whether these occurred before or after viremia detection is unknown.

### 2.3. Outcome Measurements

The primary outcomes were (i) hospitalization, ICU-level care, and all-cause mortality at 30 days and 1 year after diagnosis of CMV DNAemia, and (ii) clinical documentation of CMV end-organ disease, identified by ICD-10 code, at 30 days and 1 year after diagnosis of CMV DNAemia. Secondary outcomes were clinical factors associated with 90-day all-cause mortality in patients with CMV DNAemia. Notably, the mortality outcome assessed in this study is all-cause mortality, not CMV-attributable mortality, as the latter would require histopathological confirmation of tissue-invasive disease or clinical adjudication, both of which are not available in the TriNetX database.

### 2.4. Statistical Analysis

We completed the statistical analyses of the global federated research network using the TriNetX platform. Descriptive statistics were presented as means and standard deviations for continuous variables and as frequencies and percentages for categorical variables. Continuous data were compared using independent t-tests, whereas categorical data were compared using χ^2^ or Fisher’s exact test, as appropriate. Propensity score matching was performed using a 1:1 greedy nearest-neighbor algorithm using a caliper width of 0.1 pooled standard deviations. Balance across covariates was assessed using the standardized mean difference, and absolute values >0.1 were considered indicative of residual imbalance. Propensity score matching was conducted to balance patient demographics and comorbidities thought to be linked with increased mortality (anemia, essential hypertension, neoplasms, type 2 diabetes mellitus, and heart failure). These five covariates were selected because they represent the most prevalent comorbidities in the cohort with established independent associations with all-cause mortality. Variables such as neutropenia, glucocorticoid use, and malignancy subtype were not included in the propensity model because they were considered potential mediators on the causal pathway between immunosuppression and CMV-related outcomes, and their inclusion could introduce overadjustment bias. We also reported Bonferroni-corrected *p*-values. Additionally, the TriNetX platform’s PSM module imposes practical constraints on the number of covariates that can be simultaneously matched. We acknowledge that omitting these variables may lead to residual confounding, which is discussed as a study limitation. Additional graphs were designed using GraphPad Prism version 8.0.0 for Windows (GraphPad Software, San Diego, CA, USA, www.graphpad.com).

### 2.5. Data Access

The corresponding author had full access to the study data and was ultimately responsible for deciding to submit the manuscript for publication. The aggregated datasets generated and analyzed in the current study are available from the TriNetX platform with a subscription or through the corresponding author upon a formal, reasonable request.

### 2.6. Ethics Statement

TriNetX, LLC complies with the Health Insurance Portability and Accountability Act (HIPAA), the US federal law protecting the privacy and security of healthcare data, and any additional data privacy regulations applicable to the contributing HCO. TriNetX is certified to ISO 27001:2013 (https://trinetx.com/press-releases/trinetx-achieves-iso-270012013-certification/, accessed on 5 April 2026) and maintains an Information Security Management System to protect the healthcare data it accesses and meet the requirements of the HIPAA Security Rule. Any data displayed on the TriNetX Platform in aggregate form, or any patient-level data provided in a data set generated by the TriNetX Platform, only contains de-identified data as per the de-identification standard defined in Section §164.514(a) of the HIPAA Privacy Rule. The process of de-identifying data is attested to through a formal determination by a qualified expert as defined in Section §164.514(b)(1) of the HIPAA Privacy Rule. Geographic reporting at the regional level prevents potential re-identification by localizing patients or HCOs. TriNetX research does not require institutional review board approval because patient-identifiable information is inaccessible to users. According to the Colorado Multiple Institutional Review Board at the University of Colorado Denver, the current project follows HIPAA.

## 3. Results

### 3.1. Clinical Characteristics of NHNT Patients Who Develop CMV DNAemia

A total of 1123 patients with CMV DNAemia ≥ 5000 IU/mL were identified. The median age was 53 years. Of the cohort, 48% were male, and 52% were White. Neoplasm (63%) was the most common comorbidity, consistent with the known vulnerability of patients with hematologic malignancies to CMV reactivation [2,17]. Other commonly reported hematologic signs were purpura (55%) and neutropenia (35%). Venous thromboembolism (VTE) was present in 31% of the cohort at the time of CMV DNAemia diagnosis. Other reported signs and symptoms were generally nonspecific (malaise/fatigue [55%]; fever [53%]; dyspnea [53%]; nausea/vomiting [50%]; abdominal/pelvic pain [50%]; and cough [41%]). A high proportion of patients received glucocorticoids (91%) and anticoagulants (83%) (Supplementary Table S5).

### 3.2. Outcome Measures in NHNT Patients with CMV DNAemia

The 30-day and 1-year hospitalization rates were 35% and 37%, respectively. The 30-day and 1-year ICU level care rates were 13% and 15%, respectively. The 30-day and 1-year all-cause mortality rates were 14% and 26%, respectively (Table 1). These mortality rates are broadly consistent with prior reports of CMV DNAemia in non-allogeneic HCT cancer patients, where significant morbidity and mortality have been observed [17]. CMV esophagitis, pneumonitis, retinitis, and colitis, as identified by ICD-10 code, were documented in 11.2%, 6.0%, 3.0%, and 3.4%, respectively, within 1 year (Supplementary Table S6). The relatively low rate of documented CMV end-organ disease by ICD-10 code may reflect underdiagnosis, the absence of histopathological confirmation in many cases, or the possibility that CMV DNAemia in this population frequently represents viral replication without tissue-invasive disease.

### 3.3. Clinical Characteristics of NHNT Non-Survivors with CMV DNAemia

**Figure 1:**
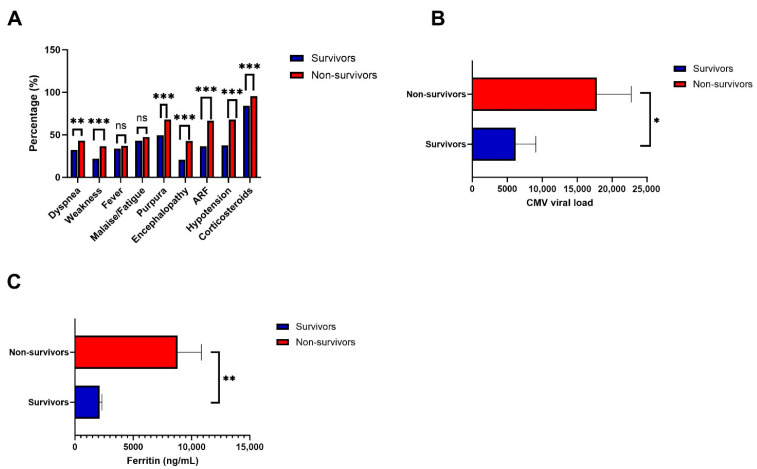
Clinical factors associated with Mortality from CMV DNAemia at 90 Days Following Propensity Score Matching: (**A**) Symptoms and comorbidities. (**B**) CMV viral load (IU/mL). (**C**) Ferritin (ng/mL). The upper bars represent the standard error. ARF: Acute respiratory failure. ns: non-significant *p*-value, * *p*-value < 0.05, ** *p*-value < 0.01, *** *p*-value < 0.001.

After matching comorbidities associated with 90-day mortality, a cohort of 556 patients was generated, with 278 non-survivors and 278 survivors. Compared with the survivor group, the non-survivor group had significantly more purpura (68% vs. 49.6%, *p* < 0.0001), dyspnea (43.2% vs. 32.4%, *p* = 0.0087), and weakness (36.7% vs. 21.9%, *p* < 0.0001) (Table 2, Figure 1A). Fever, malaise, and gastrointestinal symptoms (nausea/vomiting, aphagia/dysphagia, abdominal/pelvic pain) were not significantly different in survivor and non-survivor groups (Table 2, Figure 1A). Compared with the survivor group, the non-survivor group had significantly more malnutrition (51.4% vs. 34.5%, *p* < 0.0001), encephalopathy (42.8% vs. 20.5%, *p* 0.0001), hypotension (51.4% vs. 33.8%, *p* < 0.0001), acute respiratory failure (ARF) (66.5% vs. 36.7%, *p* 0.0001), and pleural disease (44.2% vs. 34.2%, *p* = 0.015) (Table 2, Figure 1A). VTE was observed in 26.4% of the population, but there was no significant difference between the non-survivor and survivor groups.

Compared with the survivor group, the non-survivor group was more exposed to glucocorticoids (95.3% vs. 84.2%, *p* = 0.0001), anticoagulants (92.1% vs. 80.6%, *p* = 0.0001), and benzodiazepines (89.9% vs. 74.1%, *p* = 0.0001). In contrast, the survivor group was more exposed to tacrolimus than the non-survivor group (32.4% vs. 15.5%, *p* = 0.0001). The non-survivor group had a significantly lower CD4+ cell count (190 cells/µL vs. 408 cells/µL, *p* = 0.0054) (Table 2), higher CMV viral load (17,785.1 IU/mL vs. 6220.3 IU/mL, *p* = 0.0462) (Table 2, Figure 1B), and ferritin (5654 ng/mL vs. 1710 ng/mL, *p* 0.0001) (Table 2, Figure 1C). To address the multiplicity of comparisons in Table 2, Bonferroni-corrected *p*-values were obtained (adjusted α = 0.05/40 = 0.00125) (Table 2). After correction, CMV viral load (original *p* = 0.0462) and CD4+ cell count (original *p* = 0.0054) did not survive Bonferroni correction, and these findings should be interpreted as hypothesis-generating. Readers should note that findings from the matched analysis may not fully generalize to the unmatched population, particularly to patients at the extremes of the propensity score distribution.

## 4. Discussion

Before interpreting the findings of this study, it is essential to establish a clear conceptual framework. CMV DNAemia in NHNT patients may represent: (1) a pathogenic driver of adverse outcomes through direct tissue injury and immune dysregulation; (2) a bystander marker of severe immunosuppression and critical illness; or (3) a combination of both. The retrospective, observational design of this study cannot distinguish among these possibilities. All findings reported herein should be interpreted as associations, not causal relationships. Mechanistic hypotheses discussed below are offered as context for future hypothesis-generating research and should not be construed as evidence of causality.

### 4.1. Overview of Key Findings

Our analysis revealed several clinical factors associated with 90-day all-cause mortality in patients with CMV DNAemia, including malnutrition, encephalopathy, hypotension, ARF, and pleural disease. Nonspecific signs and symptoms such as purpura, dyspnea, and weakness were significantly more prevalent in the non-survivor group. However, it is important to emphasize that these associations do not establish a causal relationship between CMV reactivation and mortality. CMV DNAemia may represent a marker of overall illness severity and immunosuppression rather than a direct cause of death, as CMV-attributable mortality typically requires confirmation through histopathological evidence of tissue-invasive disease [8,9]. The TriNetX database does not contain histopathological data, tissue biopsy results, or autopsy findings; therefore, the mortality observed in this cohort should be interpreted as all-cause mortality in the setting of CMV DNAemia, rather than CMV-attributable mortality. Also, the association between mortality and CMV DNAemia disappeared after Bonferroni correction. Commonly reported CMV end-organ diseases (esophagitis, pneumonitis, retinitis, colitis) following CMV DNAemia were infrequently documented by ICD-10 code in the NHNT population. Without adequate adjustment for baseline severity, disentangling the contribution of CMV DNAemia from the underlying disease burden is not possible.

### 4.2. Purpura and Hematologic Manifestations

The most common presentation of CMV infection in NHNT individuals is acute-onset fever, malaise, and significant lymphocytosis [2,17]. Purpura was a common finding in the cohort and was associated with mortality by propensity score matching, even after controlling for hematologic and lymphoid malignancies (commonly associated with thrombocytopenia). Several case reports describe CMV involvement in the pathogenesis of immune thrombocytopenic purpura (ITP), particularly in ITP refractory to standard therapies such as IVIG and steroids [18,19,20]. One speculative mechanism by which CMV may contribute to ITP is molecular mimicry, in which CMV-induced antibodies cross-react with platelet glycoproteins, thereby accelerating platelet destruction. However, with many cohort patients with CMV DNAemia having neoplasms, using anticoagulants, or being critically ill and at risk for disseminated intravascular coagulation, it is unclear if purpura was due to CMV reactivation, iatrogenic causes, or comorbidity-related factors—a limitation within this study. Alternatively, a common finding in CMV mononucleosis-type syndrome is an antibiotic-associated drug rash, often associated with significant lymphocytosis, which can mimic purpura [21].

### 4.3. Immunosuppression and CMV Reactivation in NHNT Patients

Our study population consisted of NHNT patients with no prior history of solid organ transplantation, bone marrow transplantation, or HIV. Despite the absence of these classic immunosuppressive conditions, a large proportion of patients who had comorbidities potentially contributing to immune dysfunction were receiving immunosuppressive medications (glucocorticoids, tacrolimus) or were critically ill (malnutrition or encephalopathy). The predominance of neoplasms (63%) in our cohort is consistent with the known vulnerability of patients with hematologic malignancies to CMV reactivation. The ECIL 7 guidelines identify patients with lymphoid malignancies, those receiving T-cell-suppressive therapy with purine analogs, and patients receiving alemtuzumab as non-HSCT patient groups at the highest risk for CMV-associated complications [8]. More recently, CMV DNAemia has been observed in patients receiving novel immunotherapies, including bispecific antibody therapy, CAR T-cell therapy, and immune checkpoint inhibitors [9,22]. In a retrospective study at a quaternary cancer center, Tay et al. found that the most frequent underlying malignancies in non-allogeneic HCT cancer patients with CMV DNAemia were B-cell lymphoproliferative disease (31%), T-cell lymphoproliferative disease (21%), chronic lymphocytic leukemia (11%), and multiple myeloma (10%), with lymphopenia, multiple cancer therapies, coinfection, and recent receipt of systemic corticosteroids commonly observed [22]. The observation that survivors had significantly higher tacrolimus exposure than non-survivors (32.4% vs. 15.5%, *p* < 0.0001) is counterintuitive, given that tacrolimus is an immunosuppressant associated with increased CMV risk. This finding likely reflects confounding by indication: tacrolimus use in this NHNT cohort may identify a subgroup of patients with autoimmune or inflammatory conditions. This association should not be interpreted as a protective effect of tacrolimus and requires further investigation in prospective studies.

Indications for immunosuppressive medications were not captured in our analysis. However, the high prevalence of glucocorticoid use (91%) in our cohort underscores the vulnerability of patients with even transient immunosuppression to CMV reactivation, which is well documented in ICU literature [4,13,21]. Critically ill patients experience impairment of host defense mechanisms through multiple pathways, including direct stimulation of viral replication by endotoxins and inflammatory cytokines, increased catecholamine levels, and immune paralysis associated with sepsis [13]. Prospective studies have demonstrated that CMV reactivation occurs in approximately 13–35% of CMV-seropositive, immunocompetent critically ill patients, with risk factors including septic shock, lower absolute lymphocyte count, high-dose steroid use, multiple blood transfusions, and prolonged ICU stay [11,21]. In a landmark prospective cohort study, Limaye et al. demonstrated that CMV viremia at any level (adjusted OR 4.3; 95% CI 1.6–11.9) and at greater than 1000 copies/mL (adjusted OR 13.9; 95% CI 3.2–60) were independently associated with hospitalization or death by 30 days in critically ill immunocompetent patients [12].

The significantly lower CD4+ cell count in the non-survivor group (190 cells/µL vs. 408 cells/µL) in our cohort further supports the role of cellular immune dysfunction in driving adverse outcomes. This finding parallels observations in the CAR T-cell literature, where persistent lymphocytopenia below 200 cells/µL has been identified as a risk factor for clinically significant CMV reactivation [9]. However, this finding should be interpreted with caution, as CD4+ testing in non-HIV patients is non-standardized and is typically ordered selectively in patients with suspected immunodeficiency or those who are clinically more ill, introducing potential ascertainment bias. Patients in the non-survivor group may have been more likely to undergo CD4+ testing because of their greater clinical severity, potentially inflating the observed difference between groups.

The interplay between CMV reactivation and immune dysfunction may be bidirectional: while immunosuppression permits CMV reactivation, CMV itself has evolved sophisticated immune evasion mechanisms that could further suppress host immunity. These include downregulation of MHC class I and II molecules, interference with NK cell recognition via viral homologs of inhibitory ligands, exploitation of the PD-L1 inhibitory signaling pathway to evade T-cell-mediated cytotoxicity, and stimulation of immunosuppressive cytokines such as IL-10 [9,23,24,25,26,27]. This bidirectional relationship may create a vicious cycle of progressive immunosuppression and uncontrolled viral replication, particularly in patients who are already critically ill, although speculative.

### 4.4. Respiratory Complications and CMV Pneumonia

Respiratory comorbidities such as ARF, pleural disease, and associated symptoms of dyspnea and (to a lesser degree) cough were significantly more common in the non-survivor group. The prominence of ARF (66.5% vs. 36.7%, *p* 0.0001) as a factor associated with mortality is notable and may reflect an overall higher burden of critical illness or CMV pneumonitis. Within our cohort, 6% of patients with CMV DNAemia were assigned an ICD-10 code for CMV pneumonitis within 1 year (Supplementary Table S2). CMV pneumonia is among the most severe manifestations of CMV disease and carries a particularly high mortality rate. In a multicenter retrospective study of 185 immunocompromised critically ill patients with CMV end-organ disease, Fernández et al. found that CMV pneumonia was the most common manifestation (62.2%) and was independently associated with higher hospital mortality (OR 2.57; 95% CI 1.13–6.03), with overall hospital mortality of 61.4% [4]. Notably, histopathological evidence was obtained in only 8.7% of patients with pneumonia in that study, highlighting the diagnostic challenges of confirming CMV pneumonia.

However, as cases of potential viral respiratory invasion in our cohort were not confirmed through histopathological assessment—which is required for definitive diagnosis of CMV pneumonia—it is unclear whether these respiratory findings were directly related to CMV tissue invasion or reflected the overall burden of critical illness. A recent study by Sadowska-Klasa et al., examining CMV DNA in bronchoalveolar lavage (BAL) samples from non-transplant patients with hematologic malignancies found CMV DNA in 13% of BAL samples, exclusively in patients with lymphoid diseases, with frequent co-pathogens including bacteria (28%), fungal antigens (36%), respiratory viruses (36%), and herpesviruses (21%) [28]. Importantly, viral load thresholds in BAL did not predict severity or outcomes, and there was no correlation between blood and BAL viral loads. These findings underscore the complexity of attributing respiratory pathology to CMV in patients with multiple potential etiologies. Furthermore, Cao et al. reported that the 30-day, 1-year, and 5-year mortality rates of CMV respiratory infection were 21.6%, 51.4%, and 69.2%, respectively, with particularly high mortality in patients with connective tissue diseases and those receiving post-chemoradiotherapy [29]. This is a study limitation.

### 4.5. Venous Thromboembolism and CMV

VTE was present in 31% of our cohort at the time of CMV DNAemia diagnosis, a notably high prevalence. Although VTE was not significantly different between survivors and non-survivors after propensity score matching, the overall prevalence warrants discussion. The association between CMV infection and VTE has been increasingly recognized. In a community prospective study of over 90,000 patients, Paran et al. demonstrated that CMV-IgM seropositivity was independently associated with VTE appearance (OR 2.49; 95% CI 1.53–4.06; *p* 0.0001) following adjustment for age, sex, and other confounders [30]. A systematic review by Ceccarelli et al. identified 115 cases of CMV-related thrombosis in immunocompetent individuals, finding that nearly half of female patients were using estrogen–progestin contraception and nearly half of all patients had an underlying coagulation disorder [31]. Proposed mechanisms for CMV-associated thrombosis include transient formation of antiphospholipid antibodies, direct endothelial cell infection leading to a procoagulant state, and CMV-induced expression of tissue factor and adhesion molecules on the endothelial surface [32,33,34,35]. However, the high VTE prevalence in our cohort must be interpreted cautiously, as many patients had neoplasms (a well-established risk factor for VTE), were receiving anticoagulants (suggesting pre-existing thrombotic risk), or had other comorbidities associated with hypercoagulability, including diabetes mellitus and critical illness. The contribution of CMV reactivation to VTE risk in this population, independent of these confounders, cannot be determined from our data.

### 4.6. Ferritin as a Biomarker in CMV DNAemia

CMV DNAemia indicates viral replication, which causes host cell cytolysis through direct lysis of infected cells and immune-mediated lysis. This may be reflected in substantially elevated ferritin levels, considered a marker of cell injury or destruction [36]. Serum ferritin has been characterized as primarily a leakage product from damaged cells, and its elevation correlates with biomarkers of cell damage, hydroxyl radical formation, and oxidative stress.

Cytolysis releases damage-associated molecular patterns (DAMPs), which likely act as potent immunosuppressors and may contribute to morbidity and mortality in this population [37]. Given the significant comorbidity burden within our cohort, both related to acute critical illness and chronic disease states, the lack of comparison of ferritin levels between cohort patients with CMV DNAemia and a control group without CMV DNAemia is a limitation of this study. Further investigation into the interaction between CMV and ferritin, including prospective studies comparing ferritin levels in patients with and without CMV DNAemia matched for underlying disease severity, is warranted to clarify whether elevated ferritin reflects CMV-specific pathology or the overall burden of critical illness.

### 4.7. Limitations

The cohort is highly heterogeneous, encompassing patients with diverse malignancies, varying degrees of critical illness, and multiple forms of immunosuppression. While this reflects real-world clinical complexity, it reduces interpretability without appropriate subgroup analyses. The propensity score model included only five covariates, and important confounders, such as severity-of-illness scores, immunosuppressive burden, neutropenia, and sepsis, were not available for matching within the TriNetX platform. Differences in antiviral prescribing patterns between survivors and non-survivors are confounded by indication and immortal-time bias and should not be interpreted as evidence of treatment efficacy or harm. Residual confounding, therefore, cannot be excluded, and the findings should be interpreted accordingly. Another major limitation of this study is the absence of a comparator group without CMV DNAemia. Without such a control group, it is not possible to determine whether CMV DNAemia independently increases risk compared with similar patients without detectable viremia. The TriNetX platform does not readily permit the construction of a well-matched control cohort, as the indication for CMV viral load testing itself introduces selection bias. This limitation restricts the ability to address whether CMV DNAemia is a causal contributor to adverse outcomes or merely a marker of underlying disease severity. The 5000 IU/mL viral load threshold used to define CMV DNAemia lacks validation across populations, and the robustness of findings at alternative thresholds has not been assessed. The inability to establish temporal relationships between CMV DNAemia and associated clinical variables is a significant limitation. Many identified associations may reflect consequences of disease progression rather than antecedent risk factors. The TriNetX database does not provide validated illness severity scores (e.g., APACHE II, SOFA), precluding adequate adjustment for baseline severity of illness. Many variables associated with mortality in this analysis—including acute respiratory failure, hypotension, encephalopathy, and malnutrition—are well-established markers of critical illness and may reflect the underlying disease burden rather than CMV-specific effects. Stratified analyses by ICU vs. non-ICU status and malignancy vs. non-malignancy subgroups were not performed due to platform constraints and anticipated small subgroup sizes. Still, they would be valuable in future studies with more granular data. These represent a limitation that may affect the generalizability of the results.

## 5. Conclusions

NHNT patients with CMV DNAemia represent a heterogeneous population with substantial comorbidity burden, particularly neoplasms, and frequent exposure to immunosuppressive medications. NHNT patients with DNAemia ≥ 5000 IU/mL are associated with high rates of hospitalization (35% at 30 days), ICU admission (13% at 30 days), and all-cause mortality (14% at 30 days; 26% at 1 year). Propensity score-matched analysis identified several clinical factors associated with 90-day all-cause mortality, including hypotension, acute respiratory failure, encephalopathy, malnutrition, elevated ferritin, and glucocorticoid exposure. VTE was notably prevalent (31%) in this cohort, although it was not differentially associated with mortality after matching.

Critically, the findings of this study should be interpreted with the understanding that CMV DNAemia does not equate to CMV disease, and the observed mortality is all-cause rather than CMV-attributable. The TriNetX database lacks the histopathological, microbiological, and clinical adjudication data necessary to confirm CMV as a direct cause of end-organ disease or death. Whether CMV DNAemia in NHNT patients represents a pathogenic driver of adverse outcomes, a bystander marker of severe immunosuppression and critical illness, or a combination of both remains an open question.

Future research should prioritize prospective studies with systematic histopathological confirmation of CMV end-organ disease, serial viral load monitoring with standardized assays, and matched control groups to determine whether CMV DNAemia independently contributes to morbidity and mortality in NHNT populations. Additionally, studies evaluating the role of CMV-specific cell-mediated immunity (e.g., CMV-specific T-cell assays) in risk stratification may help identify which NHNT patients with CMV DNAemia are at the highest risk for progression to tissue-invasive disease and may benefit from targeted antiviral intervention. Until such data are available, clinicians should maintain a high index of suspicion for CMV reactivation in NHNT patients with risk factors identified in this study—particularly those with hematologic malignancies, high-dose glucocorticoid exposure, and critical illness—while recognizing that the overall clinical context should guide the decision to initiate antiviral therapy and, when feasible, histopathological confirmation of end-organ involvement.

## Figures and Tables

**Table 1:** Analysis of outcomes at 30 and 365 days following diagnosis of CMV DNAemia (N = 1123).

Outcome	n (%)
**Hospitalization**	
30 Days	326 (35%)
365 Days	347 (37%)
**ICU Level Care**	
30 Days	119 (13%)
365 Days	142 (15%)
**Mortality**	
30 Days	134 (14%)
365 Days	240 (26%)

**Table 2:** Clinical characteristics of patients with CMV DNAemia after propensity score matching: a comparison between survivors and non-survivors at 90 days.

Variable	Total N = 556	Non-Survivor N = 278	Survivor N = 278	*p* Value
**Demographics**				
Age at CMV DNAemia * (Mean ± SD)	59.9 ± 14.8	59.5 ± 14.9	60.2 ± 14.8	0.5503
Female *	298 (53.6%)	151 (54.3%)	147 (52.9%)	0.7337
Male	258 (46.4%)	127 (45.7%)	131 (47.1%)	0.7337
Not Hispanic or Latino	409 (73.6%)	201 (72.3%)	208 (74.8%)	0.5008
White *	318 (57.2%)	157 (56.5%)	161 (57.9%)	0.7317
Hispanic or Latino *	98 (17.6%)	47 (16.9%)	51 (18.3%)	0.6562
Black or African American	61 (11%)	27 (9.7%)	34 (12.2%)	0.3422
Asian	47 (8.5%)	28 (10.1%)	19 (6.8%)	0.1700
**Comorbidities**				
Anemia *	498 (89.6%)	245 (88.1%)	253 (91%)	0.2670
Essential hypertension *	341 (61.3%)	166 (59.7%)	175 (63%)	0.4332
Neoplasms (Any) *	331 (59.5%)	165 (59.4%)	166 (59.7%)	0.9311
Hematopoietic and Lymphoid Malignancies *	207 (37.2%)	103 (37.1%)	104 (37.4%)	0.9301
Acute Respiratory Failure	287 (51.6%)	185 (66.5%)	102 (36.7%)	<0.0001
Malnutrition	239 (43%)	143 (51.4%)	96 (34.5%)	0.0001
Diseases of the Pleura	218 (39.2%)	123 (44.2%)	95 (34.2%)	0.0150 **
Neutropenia	207 (37.2%)	106 (38.1%)	101 (36.3%)	0.6609
Type 2 Diabetes Mellitus *	207 (37.2%)	102 (36.7%)	105 (37.8%)	0.7924
Unspecified Encephalopathy	176 (31.7%)	119 (42.8%)	57 (20.5%)	<0.0001
Heart Failure *	175 (31.5%)	93 (33.5%)	82 (29.5%)	0.3151
Venous thromboembolism	147 (26.4%)	83 (29.9%)	64 (23%)	0.0677
**Symptoms**				
Purpura	327 (58.8%)	189 (68%)	138 (49.6%)	<0.0001
Hypotension	237 (42.6%)	143 (51.4%)	94 (33.8%)	<0.0001
Malaise and Fatigue	252 (45.3%)	132 (47.5%)	120 (43.2%)	0.3066
Dyspnea	210 (37.8%)	120 (43.2%)	90 (32.4%)	0.0087 **
Fever, Unspecified	197 (35.4%)	103 (37.1%)	94 (33.8%)	0.4249
Nausea and Vomiting	178 (32%)	88 (31.7%)	90 (32.4%)	0.8557
Abdominal and Pelvic Pain	172 (30.9%)	81 (29.1%)	91 (32.7%)	0.3589
Weakness	163 (29.3%)	102 (36.7%)	61 (21.9%)	0.0001
Aphagia and Dysphagia	134 (24.1%)	73 (26.3%)	61 (21.9%)	0.2341
**Medications**				
Glucocorticoids	499 (89.7%)	265 (95.3%)	234 (84.2%)	<0.0001
Anticoagulants	480 (86.3%)	256 (92.1%)	224 (80.6%)	0.0001
Benzodiazepines	456 (82%)	250 (89.9%)	206 (74.1%)	<0.0001
Valganciclovir	262 (48%)	92 (33%)	170 (61%)	<0.0001
Ganciclovir	312 (56%)	192 (69%)	120 (43%)	<0.0001
Antineoplastics, other	179 (32.2%)	97 (34.9%)	82 (29.5%)	0.1733
Tacrolimus	133 (23.9%)	43 (15.5%)	90 (32.4%)	<0.0001
Antineoplastics, antimetabolites	129 (23.2%)	54 (19.4%)	75 (27%)	0.0349
**Initial Lab Values (Mean ± SD)**				
CMV Serum Viral Load (IU/mL)	12,002.7 ± 34,546.6	17,785.1 ± 43,999.8	6220.3 ± 25,093.5	0.0462 **
CD4+ Cell Count (Cells/µL)	509 ± 1606	190 ± 224	408 ± 458	0.0054 **
Leukocytes (10^3^/µL)	9.23 ± 14.6	27.5 ± 269	8.6 ± 7.53	0.0393
Neutrophils (10^3^/µL)	36.3 ± 16.3	37.4 ± 28.2	23 ± 20.6	<0.0001
Ferritin (ng/mL)	2359 ± 4092	5654 ± 15,869	1710 ± 3267	<0.0001
Bilirubin (mg/dL)	1.76 ± 4.65	5.3 ± 9.3	1.15 ± 2.94	<0.0001
Lactate Dehydrogenase (U/L)	497 ± 544	839 ± 1060	435 ± 525	<0.0001

* Variables matched in post-propensity score calculations. ** variables with a corrected Bonferroni-adjusted *p*-value ≥ 0.05.

## Data Availability

The aggregated datasets generated and analyzed in the current study are available on the TriNetX platform with a subscription or upon reasonable request from the corresponding authors.

## Supplementary Materials

The following supporting information can be downloaded at https://www.mdpi.com/article/10.3390/pathogens15050492/s1. Supplementary Materials S1: Index event definition; Supplementary Materials S2: LOINCs used for CMV viral load identification. Tables S1: International Classification of Diseases, Tenth Revision, Clinical Modification (ICD-10-CM) diagnosis codes for underlying comorbidities. Table S2: International Classification of Diseases, Tenth Revision, Clinical Modification (ICD-10-CM) diagnosis codes for symptoms. Table S3: RxNorm Codes for Medications. Table S4: Logical Observation Identifiers Names and Codes (LOINC) for laboratory values. Table S5: Characterization of clinical characteristics of patients with CMV DNAemia in HIV-Negative Non-Transplant Patients. Overall description, comparison of non-survivors vs. survivors (90 days after index event). Table S6: Additional 30 and 365-day post-CMV Disease diagnosis outcome analysis.

## Abbreviations

The following abbreviations are used in this manuscript:

Abbreviation	Definition
ARF	Acute respiratory failure
CMV	Cytomegalovirus
DAMPs	Damage-associated molecular patterns
HCO	Healthcare organization
HCT	Hematopoietic cell transplant
HIPAA	Health Insurance Portability and Accountability Act
HIV	Human immunodeficiency virus
ICU	Intensive care unit
ITP	Immune thrombocytopenic purpura
LOINC	Logical observation identifiers, names, and codes
NAAT	Nucleic acid amplification testing
NAT	Nucleic acid testing
NHNT	Non-HIV-infected, non-solid organ/bone marrow transplant
PCR	Polymerase chain reaction
VTE	Venous thromboembolism
