# Supplementary material for: Clinical Characteristics and Outcomes of Cytomegalovirus DNAemia in Non-HIV-Infected and Non-Transplant Patients: A Propensity Score-Matched Analysis
Source: Pathogens. 2026 May 1;15(5):492. doi: 10.3390/pathogens15050492 (PMC13209354; doi:10.3390/pathogens15050492)
Supplement: Supplementary file 1 [file pathogens-15-00492-s001.zip › pathogens-4273871-supplementary.pdf]

Supplementary Materials:

Supplementary S1： Index event definition

Query Criteria for the Cohort

Patients must have:

None of the following:

Human Immunodeficiency Virus (HIV) disease (USMLS:ICD10CM:B20.0); or

Transplanted organ and tissue status (USMLS:ICD10CM:Z94.0)

All of the following:

Age (Age) (at least 18 years (most recent occurrence)); and

Cytomegaloviral Disease (UMLS:ICD10CM:B25.0).

One of the following:

≥5000 copies/mL Cytomegalovirus DNA in serum, blood, plasma, tissue, or urine (USMLS:ICD10CM:30247-1,54206-8,29604-6,33006-8,53763-9,49347-8,49351-0); or

≥5000 Units/mL Cytomegalovirus DNA in serum or plasma (USMLS:ICD10CM:72493-0,34720-3,96396-7); or

Positive Cytomegalovirus DNA [Presence] in serum, plasma, blood, urine, or cerebrospinal fluid (USMLS:ICD10CM:5000-5,30246-3,30326-3,4996-5,4999-9,4998-1)

Supplementary S2： LOINCs used for CMV viral load identification

Primary Outcome Analysis

Non-Survivors

Patients must have:

any of the following:

Deceased (Deceased); or

Ill-defined and unknown cause of mortality (UMLS:ICD10CM:R99).

Survivors

Patients must not have:

any of the following:

Deceased (Deceased); or

Ill-defined and unknown cause of mortality (UMLS:ICD10CM:R99).

Table S1. International Classification of Diseases, Tenth Revision, Clinical Modification (ICD-10-CM) diagnosis codes for underlying comorbidities.

Code	Characteristic Name
AI	Age at Index
2186-5	Not Hispanic or Latino
2106-3	White
M	Male
F	Female
2054-5	Black or African American
2131-1	Unknown Race
2135-2	Hispanic or Latino
2028-9	Asian
B20	HIV Disease
C00-D49	Neoplasms
C81-C96	Malignant neoplasms of lymphoid, hematopoietic and related tissue
D60-D64	Aplastic and other anemias and other bone marrow failure syndromes
D69	Purpura and other hemorrhagic conditions
D70	Neutropenia
D80	Immunodeficiency with predominantly antibody defects
E11	Type 2 diabetes mellitus
E23	Hypopituitarism and other disorders of the pituitary gland
E27	Other disorders of adrenal gland
E40-E46	Malnutrition
E87.1	Hypo-osmolality and hyponatremia
E87.6	Hypokalemia
F20-F29	Schizophrenia, schizotypal, delusional, and other non-mood psychotic disorders
F41	Other anxiety disorders
G93.4	Other and unspecified encephalopathy
I10	Essential (primary) Hypertension

I50	Heart Failure
I82	Other venous embolism and thrombosis
I95	Hypotension
J40-J47	Chronic lower respiratory diseases
J90-J94	Other diseases of the pleura
J96.0	Acute respiratory failure
K50-K52	Noninfective enteritis and colitis
M30-M36	Systemic connective tissue disorders
Z94.81	Bone Marrow Transplant Status

Table S2. International Classification of Diseases, Tenth Revision, Clinical Modification (ICD-10-CM) diagnosis codes for symptoms.

**Code**	**Symptom**
R00	Abnormalities of heart beat
R05	Cough
R06.0	Dyspnea
R07	Pain in throat and chest
R10	Abdominal and pelvic pain
R11	Nausea and vomiting
R13	Aphagia and dysphagia
R50	Fever of other and unknown origin
R50.9	Fever, unspecified
R53	Malaise and fatigue
R53.1	Weakness
R60	Edema, not elsewhere classified

Table S3. RxNorm Codes for Medications.

Code	Medication
AN300	Antineoplastics, antimetabolites
AN900	Antineoplastics, other
BL110	Anticoagulants
CN302	Benzodiazepine derivative sedatives
CV702	Loop diuretics
HS050	Adrenal corticosteroids
HS051	Glucocorticoids
HS700	Pituitary
OP210	Antibacterials, topical ophthalmic
OP300	Anti-inflammatories, topical ophthalmic
RE102	Bronchodilators, sympathomimetic
5492	Hydrocortisone
42316	Tacrolimus
68149	Mycophenolate mofetil

Table S4. Logical Observation Identifiers Names and Codes (LOINC) for laboratory values

Code	Laboratory Test
9008	Erythrocyte distribution width [Ratio]
9011	Erythrocyte mean corpuscular volume
9015	Leukocytes [#/volume] in Blood
9016	Lymphocytes/100 leukocytes in Blood
9017	Monocytes/100 leukocytes in Blood
9018	Neutrophils [#/volume] in Blood
9022	Calcium [Mass/volume] in Serum
9031	Activated partial thromboplastin time
9032	INR
9033	Prothrombin time
9035	Cortisol [Mass/volume] in Serum
9040	Thyrotropin [Units/volume] in Serum
9042	Ferritin [Mass/volume] in Serum
9050	Billirubin total [Mass/volume] in Serum
9052	Lactate dehydrogenase [Enzymatic activity/volume] in Serum

Table S5. Characterization of clinical characteristics of patients with CMV DNAemia in HIV-Negative Non-Transplant Patients. Overall description, comparison of non-survivors vs. survivors (90 days after index event).

Variable<br>Mean ± SD, N (%)	Overall,<br>N = 1123	Non-survivors,<br>N = 234	Survivors<br>N = 889	P-value
**Demographics**				
Age at Index	53 ± 18	65.1 ± 15.3	56.6 ± 18.2	< 0.0001
Male	543 (48%)	106 (45%)	437 (49%)	0.2867
Female	579 (52%)	128 (55%)	451 (51%)	0.2867
White	579 (52%)	142 (61%)	516 (58%)	0.4766
Hispanic or Latino	253 (23%)	41 (18%)	212 (24%)	0.0386
Not Hispanic or Latino	788 (70%)	166 (71%)	622 (70%)	0.7899
Black or African American	104 (9%)	20 (9%)	84 (9%)	0.6685
Asian	85 (8%)	20 (9%)	65 (7%)	0.528
**Comorbidities**				
Aplastic Anemia	894 (80%)	213 (91%)	681 (77%)	< 0.0001
Neoplasms	710 (63%)	159 (68%)	551 (62%)	0.0958
Essential Hypertension	662 (59%)	154 (66%)	508 (57%)	0.0173
Purpura	619 (55%)	171 (73%)	448 (50%)	< 0.0001
Acute Respiratory Failure	480 (43%)	171 (73%)	309 (35%)	< 0.0001
Malnutrition	452 (40%)	127 (54%)	325 (37%)	< 0.0001
Pleural Diseases	444 (40%)	122 (52%)	322 (36%)	< 0.0001
Hypotension	436 (39%)	128 (55%)	308 (35%)	< 0.0001
Neutropenia	397 (35%)	93 (40%)	304 (34%)	0.1169
Type 2 Diabetes Mellitus	394 (35%)	95 (41%)	299 (34%)	0.0483
Sepsis	390 (35%)	159 (68%)	231 (26%)	< 0.0001
Lymphoid Malignancies	369 (33%)	85 (36%)	284 (32%)	0.2084

Venous Embolism and Thrombosis	343 (31%)	86 (37%)	257 (29%)	0.021
Other and Unspecified Encephalopathy	310 (28%)	109 (47%)	201 (23%)	< 0.0001
Heart Failure	292 (26%)	93 (40%)	199 (22%)	< 0.0001
**Symptoms**				
Malaise and Fatigue	623 (55%)	134 (57%)	489 (55%)	0.5473
Fever, Unspecified	600 (53%)	111 (47%)	489 (55%)	0.0253
Dyspnea	592 (53%)	130 (56%)	462 (52%)	0.3361
Nausea and Vomiting	561 (50%)	99 (42%)	462 (52%)	0.0082
Abdominal and Pelvic Pain	557 (50%)	102 (44%)	455 (51%)	0.0374
Cough	458 (41%)	82 (35%)	376 (42%)	0.0433
Weakness	444 (40%)	110 (47%)	334 (38%)	0.0089
Aphagia and Dysphagia	289 (26%)	81 (35%)	208 (23%)	0.0005
**Medications**				
Anticoagulants	931 (83%)	214 (91%)	717 (84%)	0.0044
Antineoplastics, antimetabolites	348 (31%)	58 (25%)	290 (33%)	0.0206
Antineoplastics, other	387 (34%)	92 (39%)	295 (33%)	0.081
Antivirals	640 (57%)	222 (90%)	584 (80%)	0.0537
Benzodiazepines	946 (84%)	214 (91%)	732 (82%)	0.0007
Bronchodilators	839 (75%)	201 (86%)	638 (72%)	<0.0001
Glucocorticoids	1023 (91%)	225 (96%)	798 (90%)	0.0025
Hydrocortisone	658 (59%)	159 (68%)	499 (56%)	0.0012
Loop Diuretics	747 (67%)	196 (84%)	551 (62%)	<0.0001
Pituitary (Peptide Hormones)	267 (24%)	115 (49%)	152 (17%)	<0.0001
Tacrolimus	270 (24%)	32 (14%)	238 (27%)	<0.0001
**Lab Values**				
CMV Serum Viral Load (IU/mL)	31,236 ± 253,512	28,570 ± 133,784	14,855 ± 110,337	0.7763
CD4+ T Cell Count (Cells/mL)	509 ± 1,606	190 ± 224	408 ± 458	0.0054

Leukocytes (10^3 Cells/μL)	9.23 ± 14.6	27.5 ± 269	8.6 ± 7.53	0.0393
Neutrophils (10^3 Cells/μL)	581 ± 2,621	257 ± 2074	579 ± 2409	0.0782
aPTT	36.3 ± 16.3	37.4 ± 28.2	23 ± 20.6	< 0.0001
INR	1.33 ± 1.25	1.88 ± 4.86	1.29 ± 1.26	0.0021
PT	14.9 ± 3.98	17.4 ± 11.5	14.9 ± 6.39	< 0.0001
Ferritin (mg/dL)	2,359 ± 4,092	5654 ± 15869	1710 ± 3267	< 0.0001
Bilirubin (mg/dL)	1.76 ± 4.65	5.3 ± 9.3	1.15 ± 2.94	< 0.0001
Lactate Dehydrogenase (U/L)	497 ± 544	839 ± 1060	435 ± 525	<0.0001

Table S6. Additional 30 and 365-day post-CMV Disease diagnosis outcomes analysis.

Outcome, N = 1123		N (%)
**Esophagitis**		
	30 Days	75 (6.6%)
	365 Days	127 (11.2%)
**Pneumonitis**		
	30 Days	40 (3.4%)
	365 Days	67 (6.0%)
**Retinitis**		
	30 Days	24 (2.1%)
	365 Days	34 (3.0%)
**Pericarditis**		
	30 Days	22 (2.0%)
	365 Days	61 (5.5%)
**Colitis**		
	30 Days	21 (1.9%)
	365 Days	38 (3.4%)
